# Interrelationship of Preschoolers’ Gross Motor Skills, Digital Game Addiction Tendency, and Parents’ Parenting Styles

**DOI:** 10.3390/children12070932

**Published:** 2025-07-16

**Authors:** Savaş Aydın, Ramazan Sak, İkbal Tuba Şahin-Sak

**Affiliations:** 1 Sports Management, Faculty of Sport Sciences, Van Yüzüncü Yıl University, Van 65080, Türkiye; savasaydin@yyu.edu.tr; 2 Early Childhood Education, Faculty of Education, Van Yüzüncü Yıl University, Van 65080, Türkiye; ramazansak@yyu.edu.tr

**Keywords:** gross motor skills, digital game addiction tendency, parenting styles, preschoolers

## Abstract

Background: Motor performance in childhood predicts physical fitness, cognitive capacity, socio-emotional development, and academic success. Parenting styles are especially important to such performance in the preschool period, as children’s gross motor abilities are shaped in part by their interactions with parents. Young children’s physical activity is also declining as they spend more time on screens. Methods: This quantitative survey-based study examined the relationships among 252 preschoolers’ gross motor skills, their tendency to become addicted to digital games, and their parents’ parenting styles. Results: The sampled preschoolers’ gross motor skill development and game addiction tendencies were both low, while the participating parents reported high levels of democratic and overprotective parenting attitudes, low levels of authoritarian ones, and moderate levels of permissive ones. Motor skills were not associated with children’s addiction tendency or parents’ democratic (also known as authoritative), authoritarian, or permissive styles. However, overprotective parenting was positively and significantly associated with gross motor skill scores. While no significant relationship was found between children’s digital game addiction tendencies and their parents’ adoption of a democratic parenting style, such tendencies were positively and statistically correlated with the authoritarian and permissive parenting styles. One dimension of such tendencies, constant gameplay, was also positively and significantly correlated with overprotective parenting. Conclusions: Although the participating children’s digital game addiction tendencies were low, the findings indicate that parents and carers should guide children to reduce their screen time and promote increased interaction with their surroundings and other people to mitigate screen time’s known negative effects on gross motor coordination.

## 1. Introduction

Early childhood development is an important part of life that includes children’s physical, cognitive, motor, linguistic, social, and emotional growth [1]. During the preschool years, alongside physical growth, the brain undergoes dynamic development, and language acquisition accelerates [2]. However, it is of the utmost importance that children have experiences related to each developmental area, and if such experiences are absent from their early years, subsequent exposure to them will result in diminished benefit, or even none at all [3]. An important instance of this phenomenon is the early childhood development of motor skills [4,5,6,7]. A critical aspect of child development [8], such skills act as vital catalysts for executive functions, prosocial behaviors, and cognitive capacities [9,10], among other developmental domains [11,12,13]. Childhood motor performance forecasts future results in physical fitness [14,15], cognitive capability [16,17], socio-emotional development [16,18], and academic success [19,20]. In brief, motor performance has a pivotal impact on various aspects of a child’s future development.

Motor skills represent the synthesis of various motions, with gross motor skills (GMSs) constituting one of their fundamental components. GMSs pertain to the large, force-generating muscles of the limbs and torso being utilized to perform purposeful actions [21]. They encompass locomotor skills like running, hopping, skipping, leaping, and sliding, as well as object controlling skills such as striking, dribbling, kicking, catching, and throwing [22,23]. Cultivated in the early years, GMSs are foundational to the more sophisticated motor skills that normally develop later [24]. The specific executive functions most closely associated with GMSs are inhibitory control, working memory, and self-regulation, and substantial positive correlations have been reported between GMSs and reaction speeds during preschool-aged children’s inhibitory control activities [25,26,27].

There has been a worrying decline in children’s motor skills over the past few decades [28,29,30,31]. Amid the integration of digital technology into a growing range of everyday-life activities, today’s children engage with digital games more frequently than prior generations did, and the influence of such games on their behavior is a subject of intense research interest. However, the results of such research have been inconclusive. Video games’ reported adverse effects include heightened aggression, among other psychological, social, and physical difficulties, but they have also been hailed as enhancing attention [32], hand–eye coordination, self-esteem, intellectual functioning, and academic achievement [33,34,35]. Similarly, while excessive engagement in gaming can hinder social interactions [36], those games that necessitate collaboration can enhance players’ social competencies [37]. Discrepancies in research outcomes about the impact of digital games on children may stem from various factors including the age cohort and other demographic characteristics of the research subjects, studies’ cultural contexts, the methodologies employed for data collection, and fundamental differences among the games being played. Researchers do, however, concur that computer games influence preschool children’s behavior [38,39].

Regarding inter-game differences in particular, prolonged engagement with non-educational digital games has been found to adversely impact various domains of children’s development [40]. Educational ones, however, seem to achieve their goals of fostering learning and creativity [41], and thereby augment motivation [42,43], cognitive abilities [44,45,46], language development [47], and mathematical and other academic competencies [48,49,50].

Many individuals, including preschoolers [51], become gaming addicts [52,53,54], i.e., their excessive, uncontrollable engagement with games causes emotional and social issues, including the disruption of everyday responsibilities [55]. In preschoolers, Bağatarhan [51] found that the propensity for digital gaming addiction was negatively correlated with self-regulation skills and social competency. Bağcı-Çetin [56], meanwhile, concluded that self-regulation and such propensity in five- to six-year-old children jointly predicted their prosocial behavior. More recently, Karaca et al. [57] found that digital game addiction was negatively associated with positive peer play behaviors.

Preschool children’s physical activity levels are also falling [58] as they spend more time on screen-based activities [59]. The GMSs of preschoolers residing in metropolitan areas are significantly and negatively affected by sedentary screen usage [60]. However, screen time has been reported both as negatively correlated with the development of GMSs in children under age seven [61,62,63] and uncorrelated with it [64].

Regardless of their parenting style, parents express strong concern about technology’s impact on their children [65]. Several prior studies have examined correlations between parenting and preschoolers’ addictive inclinations towards digital games [66]. Parental “phubbing”, the act of prioritizing virtual engagement above face-to-face communication by using a smartphone during social interactions, has been found to correlate positively with such inclinations in their children, while also detracting from the latter’s development of social skills [67]. Other aspects of parenting behavior have also been linked to preschoolers’ gaming addiction [68,69]. Notably, Keya et al. [70] and Akaroğlu [71], respectively, reported that permissive and authoritarian parenting styles predicted such addiction.

Children’s interactions with their parents and siblings and their activities within the home profoundly influence their GMSs [8], and parenting styles are particularly important in this regard during the preschool years [72,73]. However, this is not a case of direct causation, but is influenced by the environmental support, opportunities, and emotional atmosphere that parents offer. This study therefore adopted Bronfenbrenner’s ecosystem theory as its fundamental framework for elucidating this process. This theory analyzes multiple environmental layers—i.e., microsystem, mesosystem, exosystem, and macrosystem—that influence child development [74,75]. Parenting styles reside in the microsystem, the child’s immediate home environment, but interact with other systems. The microsystem is influenced by such styles, which also directly shape children’s everyday interactions and opportunities at home. A nurturing home setting is likely to promote physical engagement via a range of motor activities and ensure a secure space for exploration. The mesosystem, encompassing school–family interaction, also influences motor skill development through parental interest in and support for their children’s engagement in physical activities at school and in playgroups. Thus, parenting styles can reasonably be expected to significantly influence both the quantity and the quality of children’s opportunities for physical activity. More specifically, children with democratic (also known as authoritative) and permissive parents exhibit better motor skills development than those with authoritarian parents. Moreover, of those three styles, the democratic one was associated with children’s highest motivation to move, and the authoritarian one with the lowest such motivation [76]. Lu et al. [6], Nadia et al. [72], and Wigati [77] all reported associations between democratic parenting and locomotor skills.

The prior literature further indicates that parenting styles may influence preschool children’s GMS levels and digital game addiction tendencies. Moreover, children today are beginning to spend more time on digital games from preschool age, and are less likely to move in parallel with this case. Consequently, it is justifiable to examine preschool children’s GMSs, their parents’ parenting styles, and their digital game addiction tendencies concurrently. Nevertheless, no prior work appears to have done so. The purpose of this study is therefore to fill this gap, guided by the following three research questions (RQs).

RQ1. Is there a significant relationship between preschoolers’ GMS levels and their digital game addiction tendency?

RQ2. Is there a significant relationship between preschoolers’ GMS levels and the parenting styles of their parents?

RQ3. Is there a significant relationship between preschoolers’ digital game addiction tendency and the parenting styles of their parents?

## 2. Materials and Methods

### 2.1. Research Design

This study employed a quantitative survey-based methodology, which is appropriate for the assessment of the current state of a certain group’s abilities, views, attitudes, beliefs, and knowledge [78]. Data were collected from a random sample of the Turkish preschool population, using established scales for each of the three constructs of interest.

### 2.2. Participants

The participants comprised 252 children attending preschool institutions affiliated with the Van Provincial Directorate of National Education, and one of each of those children’s parents. The criteria for participant selection were as follows. Preschool children had to be enrolled in a preschool of the type noted above and exhibit normal developmental progress. Parents had to have a normally developing child enrolled in a preschool education facility. From among the 85 such institutions in the city center of Van, 10 were chosen at random. Then, one class was chosen, also at random, from among all classes in each of those 10 institutions; and 10 children and their families were randomly selected from each of those classes and informed about the study. Data from 252 children who assented to participate, whose parents consented to their participation, and who completed the data collection instruments accurately were analyzed. Further information about the participants is provided in Table 1.

### 2.3. Data Collection Tools

#### 2.3.1. Demographic Information Form

A Demographic Information Form created by the researchers was used to capture the categories of descriptive and demographic information about the children and their parents that are set forth in Table 1.

#### 2.3.2. Parenting Style Scale

This 46-item tool, developed by Karabulut Demir and Şendil [79] based in part on earlier work by Baumrind [80] and Maccoby and Martin [81], measures the child rearing attitudes of parents with children aged two to six. It has four sub-dimensions, i.e., authoritative (i.e., democratic; 11 items), authoritarian (17 items), permissive (9 items), and overprotective (9 items), all answered on the same Likert-type scale with the options 1 = “never”, 2 = “rarely”, 3 = “sometimes”, 4 = “usually”, and 5 = “always”.

The democratic parenting style involves accepting that the child is a separate, independent person/personality and encouraging them to express their ideas. The authoritarian style, in contrast, implies little acceptance that the child is a separate individual. Its associated practices include single-sided communication, pressure, unconditional obedience to rules, and verbal and physical punishment. Parents with an overprotective style, meanwhile, believe that the child must constantly be protected, and is marked by reluctance to accord the child meaningful responsibility. Lastly, the permissive style implies welcoming everything the child does, granting them too much freedom, and in common parlance spoiling them.

There are no reverse-coded items in the Parenting Style Scale, and the scores obtained from each of its dimensions are calculated separately, with the highest-scoring dimension indicating the participant’s parenting style [79]. Cronbach’s alpha reliability values were computed by its originators as 0.74, 0.75, 0.76, and 0.83 for the permissive, overprotective, authoritarian, and authoritative dimensions, respectively. The present authors further established that the Cronbach’s alpha for the whole scale was 0.86.

#### 2.3.3. TGMD-2

GMSs were assessed using the Test for Gross Motor Development, 2nd edition (TGMD-2), widely regarded as the gold standard for this type of evaluation, with exceptional test–retest reliability [82], including for children in Türkiye [83]. It measures 12 GMSs comprising six locomotor skills (running, galloping, jumping, sliding, leaping, and hopping) and six object control skills (striking, stationary dribbling, kicking, catching, overhand throwing, and underhand rolling of a ball) [84]. The test is administered to children individually. After the administrator illustrates the proper form and process of each of the 12 GMSs, the participant executes each skill twice, and is scored based on the presence (1) or absence (0) of each scoring criterion. The scores from each skill are then aggregated to create subtest scores for locomotor skills and object control skills. This study adopted the standard approach with this instrument [82] of evaluating scores of 89 and below as low, scores between 90 and 120 as medium and scores of 121 and above as high. This study’s authors computed the Cronbach’s alpha of the TGMD-2 as used here as 0.85.

#### 2.3.4. DGAT

The 20-item Digital Game Addiction Scale (DGAT) was developed by Budak and Işıkoğlu [85] to ascertain preschool children’s tendency to become addicted to digital games. It consists of four dimensions: dissociation from life (7 items), conflict (5 items), constant play (5 items), and reflection on life (3 items). The first covers the extent to which children distance themselves from their social lives while playing digital games. The conflict dimension reflects their reactions when the process of playing digital games is restricted or prevented. Constant play covers the length of time a child plays games and how often they demand to do so; reflection on life captures the effects of digital games on the child’s daily life, including any problematic symptoms. The scale has a five-point Likert-type structure with ratings ranging from 1 = “never” to 5 = “always”. The lowest possible score is therefore 20, and the highest, 100. Exploratory factor analysis of the construct validity of this scale revealed that the four-factor structure accounted for slightly more than 63% of total variance. Confirmatory factor analysis indicated that its model fit values were good (χ^2^/sd = 3.402, RMSEA = 0.075, AGFI = 0.85, CFI = 0.92, IFI = 0.92, SRMR = 0.52, PNFI = 0.76, PGFI = 0.68). The dimensions can be evaluated separately or collectively, with higher scores in either case indicating a greater tendency towards addiction [85]. The present authors’ computation of the Cronbach’s alpha for the whole DGAT-2 was 0.95.

### 2.4. Data Collection Procedure

After receiving ethical permission from their university’s ethics board, the researchers compiled a list of all Ministry of National Education-affiliated preschool establishments in the target province. From within that list, schools, their classrooms, and individual children within those classes were randomly selected for participation as described above, and the purposes of the research were communicated to the relevant instructors as well as the selected children’s parents. Those parents who volunteered to participate first completed the DGAT-2 on behalf of their children and the Parenting Style Scale for themselves. Later, an appropriate environment for the TGMD-2 test was prepared by the researchers. Participants awaiting their trials stayed in the classroom and engaged in their customary activities. Before the measurements were taken, as explained in the preceding subsection, the children were asked by the researchers whether they were experiencing any discomfort. In cases where the researchers were uncertain whether a child had met a performance criterion, that child was asked to perform the action a third time, and scored with reference to that criterion only. The administration of the TGMD-2 took 25–30 min per child. To establish inter-rater reliability, consistency measures were conducted using data from 12 children, who were excluded from the study prior to the collection of the main data.

### 2.5. Data Analysis

The study data were analyzed using software. The results of Kolmogorov–Smirnov testing, along with skewness and kurtosis values, suggested a non-normal data distribution. Consequently, non-parametric Spearman rho analysis was adopted.

## 3. Results

### 3.1. General

The sampled children’s GMS levels and digital game addiction tendencies, and the sampled parents’ parenting styles, are set forth in Table 2.

The children’s mean scores for locomotor skills (M = 8.77, SD = 4.42) and object control (M = 7.69, SD = 4.19) reflect low performance levels, and their overall TGMD-2 score (M = 16.30, SD = 7.21) can be regarded as indicating low gross motor development. Their mean tendency to digital game addiction, meanwhile, was also rated as low (M = 47.77, SD = 18.68). The participating parents exhibited high levels of democratic and overprotective parenting, low levels of authoritarian parenting, and medium levels of permissive parenting.

### 3.2. RQ1: Relationship Between Gross Motor Skills and Digital Game Addiction Tendency

The detailed results of Spearman’s rho analysis conducted to ascertain whether there was a significant relationship between the sampled preschool children’s GMSs and their game addiction tendency can be seen in Table 3.

**Figure 1:**
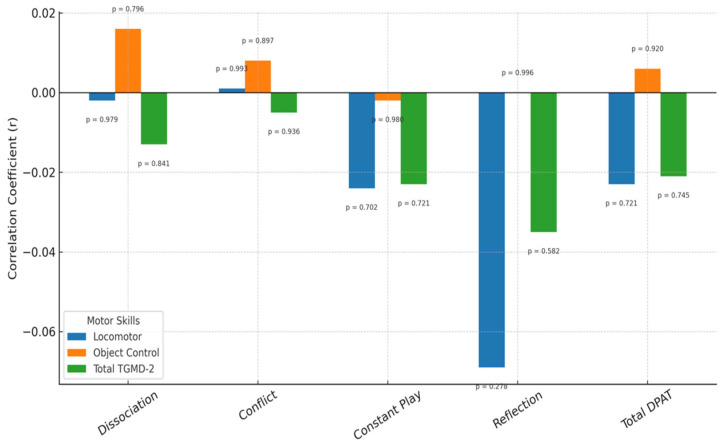
Non-significant relationship between preschool children’s gross motor skills and their digital game addiction tendency.

As this table indicates, no statistically significant relationships were found between, on the one hand, any TGMD-2 sub-dimension score or the total TGMD-2 score, and on the other, any sub-dimension of the DGAT or its total score. This relationship can be seen in Figure 1.

### 3.3. RQ2: Relationship Between Gross Motor Skills and Parenting Styles

Spearman’s rho results regarding the potential significance of the relationship between the sampled children’s GMSs and the parenting styles employed by their parents can be found in Table 4.

**Figure 2:**
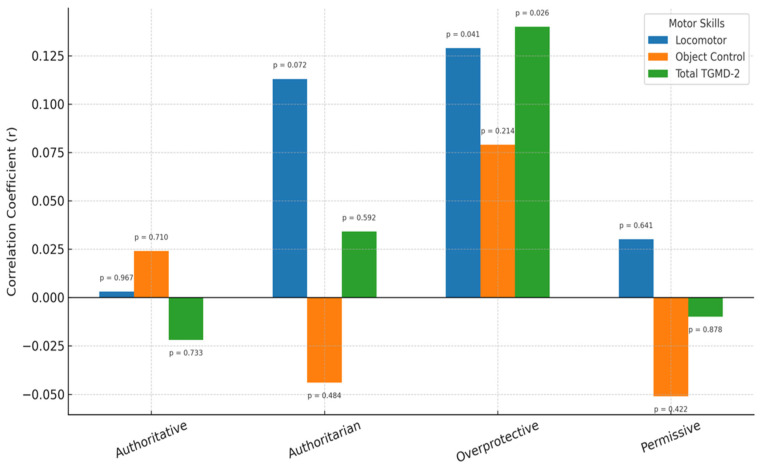
Relationships between preschool children’s gross motor skills and the parenting styles of their parents.

No significant relationship was found between the sampled parents’ democratic, authoritarian, or permissive parenting styles, on the one hand, and on the other, their children’s locomotor skills, object control skills, or overall GMS scores (*p* > 0.05). However, a medium-sized positive and significant relationship was identified between the overprotective parenting style and children’s locomotor skills score (r = 0.129, *p* = 0.041), and a small positive and significant relationship between that style and overall GMS score (r = 0.140, *p* = 0.026). In other words, it appeared that the more overprotective a parent is, the higher their children’s GMSs. This relationship is illustrated in Figure 2.

### 3.4. RQ3: Relationship Between Digital Game Addiction Tendency and Parenting Styles

The results of the Spearman rho tests of whether there was a significant relationship between the sampled preschool children’s tendency to become addicted to digital games and the parenting styles employed by their parents can be seen in Table 5.

As the table indicates, no significant relationship was found between the sampled children’s addiction tendency and the preference for an authoritative parenting style in their households. However, the results indicated a small positive and statistically significant relationship between the authoritarian parenting style, on the one hand, and on the other, overall addiction (r = 0.280, *p* = 0.001) and all four addiction sub-dimensions (dissociation, r = 0.278, *p* < 0.001; conflict, r = 0.223, *p* < 0.001; constant play, r = 0.251, *p* < 0.001; reflection, r = 0.217, *p* = 0.001).

Small significant and positive associations were also identified between the permissive parenting style and overall digital game addiction (r = 0.175, *p* = 0.005) and all four addiction sub-dimensions (dissociation, r = 0.143, *p* < 0.05; conflict, r = 0.132, *p* < 0.05; constant play, r = 0.156, *p* < 0.05; reflection, r = 0.225, *p* = 0.001).

**Figure 3:**
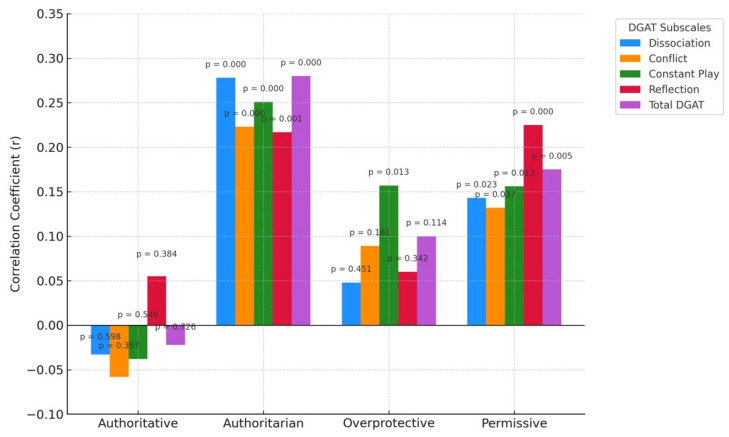
Relationships between preschool children’s digital game addiction tendency and the parenting styles of their parents.

Finally, a small positive and statistically significant correlation was found between the overprotective parenting style and one addiction sub-dimension, constant play (r = 0.157, *p* < 0.05). These relationships are illustrated in Figure 3.

## 4. Discussion

The present study’s finding that the participating children’s GMS development was at a low level contrasts with those of previous studies [86,87,88]. This could have been due to parents in its urban target region not providing their children with sufficient support for their GMS development. Factors pertinent to that phenomenon could have included the long hours parents spend working and apartments’ limited space for children to run, jump, and so forth. Additionally, parental support has been found to correlate highly with children’s motor skills [89], and 87.3% of mothers and 85.7% of fathers in this study were not actively involved in sports.

The participating children’s digital game addiction tendencies were also measured as low, echoing prior findings by Şenol et al. [90]. In early childhood, it is widely agreed to be undesirable for children to spend large amounts of time playing digital games or being exposed to screens generally. Therefore, this finding may relate to the participating parents’ awareness of their children’s digital game playing and other screen time, and efforts to limit such time. Parents’ knowledge about digital technology, together with their role modeling of exposure to it, has previously been found to exert considerable effects. Türen and Bağçeli Kahraman [91], for example, recently reported substantial correlations between mothers’ knowledge of digital parenting and their children’s inclinations toward digital game addiction, as well as between mothers’ digital literacy and their awareness of digital parenting.

The participating parents expressed high levels of democratic and overprotective parenting attitudes, low levels of authoritarian parenting attitudes, and moderate levels of permissive parenting attitudes. Those findings accord fairly well with those of Yıldız and Sak [92] and Yalçın [93], who found that among Turkish parents, the democratic and overprotective styles were dominant. However, the parents sampled in the present work had a somewhat elevated incidence of the democratic style, which may be related to their educational attainment, i.e., most were high-school or university graduates. Mızrakçı [94] concluded from his research on the determinants of maternal child rearing methods that the primary one was the educational attainment of both parents, and emphasized an observed positive correlation between the incidence of democratic parenting and parental educational attainment.

The lack of any statistically significant relationship between the current study’s sampled preschool children’s GMSs and their digital game addiction tendency was unexpected, given that in the preschool period, such motor skills develop rapidly, and excessive screen time could reasonably be expected to hinder that development process. On the other hand, most preschoolers are naturally bouncy and active, and this natural mobility might have offset the presumed negative effects of digital games on GMS scores. In any case, this result should be interpreted with caution, given that the child participants’ digital game addiction tendencies and GMS levels were both low.

The present study’s findings of no significant relationship between GMS levels and the democratic, authoritarian, or permissive parenting styles were contrary to the researchers’ expectations. Likewise, they did not expect to find a positive and significant relationships between both locomotor skills and overall GMS, on the one hand, and overprotective parenting, on the other, as it has traditionally been thought that overprotective parenting negatively affects children’s independence, and therefore their motor skills. This latter unexpected finding could have been because the overprotective parents, despite worrying about their children’s safety, permitted physical exploration in secure, regulated situations instead of entirely prohibiting it [92]: for instance, they may have established play spaces that reduce the likelihood of falls, or were present to intervene during activities. Be that as it may, the unforeseen positive correlation indicates that overprotective parenting does not invariably yield adverse outcomes and may even, in certain circumstances, enhance children’s motor skills. The reasons for this interesting finding can be examined in depth in further studies. In particular, qualitative studies could examine how overprotective parents support their children’s motor skills through interviews and observations.

The prior literature has reported significant correlations between parenting and digital game addiction tendencies [71,95]. Parents who adopt the democratic style foster their children’s independence while establishing explicit boundaries and rules [92]. However, the present study identified no significant relationship between addictive tendencies in the sampled preschool children and the democratic parenting style that predominates among better-educated Turkish parents, as noted above. This finding is not consistent with some studies [95,96]. Liu et al. [95] reported a negative correlation between democratic parenting and Internet addiction, and Suherman [96] concluded that parents ought to adopt that parenting style to mitigate device addiction in their preschool-aged children. The offspring of democratic parents, who provide constant supervision and support, were also found in the same two studies to be less prone to hazardous Internet usage tendencies. It is possible that the transparent communication, collaboration, promotion of alternative activities, and positive role models inherent in this parenting style contributed to the low incidence of digital game addiction among the preschool children sampled in the present work, and that this resulted in the lack of a significant correlation. In other words, the framework of the democratic approach, which fosters autonomy while imposing boundaries, could have facilitated the cultivation of good digital gaming habits.

The more expected findings of the present study included the positive and statistically significant relationships between authoritarian parenting and children’s digital game addiction tendencies, which closely echoed Akaroğlu’s [71], Özgür’s [97], and Liu et al.’s [95]. The authoritarian parenting style is typically defined by high expectations, stringent regulations, and minimal emotional support. These features can result in adverse emotional and social consequences for preschoolers, including stress, anxiety, diminished autonomy, and social isolation [92]. It is reasonable to expect that children of authoritarian parents will resort to digital games to mitigate these negative feelings and enhance their self-esteem, thus elevating their risk of addiction. Moreover, certain demographic and cultural traits of parents may influence the intensity of the correlation between this parenting approach and digital gaming addiction.

The current study’s finding of a positive and statistically significant relationship between permissive parenting and children’s digital game addiction tendencies echoes Keya et al.’s [70] and Liu et al.’s [95]. Permissive parents tend to establish few rules for their children and enforce those they do establish only some of the time. There is no strong reason to suppose that this pattern does not also apply to digital games, a sphere where the insufficient delineation of permissible durations and types of gaming may result in excessive time spent on this activity, heightening the risk of addiction. The other defining traits of this parenting style, including insufficient supervision, reluctance to refuse requests, failure to promote alternative activities, and generally minimal parental engagement [92], may also lead preschool children to develop excessive attachment to digital games, further heightening that risk.

Lastly, the overprotective parenting style was found to have a statistically significant (positive) relationship with only one sub-dimension of digital game addiction tendency, i.e., constant play. Overprotective parents focus on protecting their children from potential hazards and adverse experiences, and this may restrict the latter’s opportunities to engage with outdoor environments, to learn to take risks, and to have autonomous experiences [92]. In particular, the restriction of children’s engagement with non-home environments by parents with this profile could be expected to heighten the children’s desire for novel experiences within the home, culminating in incessant digital play.

## 5. Conclusions

The sampled children’s mean scores for gross motor development were low, and their mean tendency to digital game addiction was also rated as low. The participating parents exhibited high levels of democratic and overprotective parenting, low levels of authoritarian parenting, and medium levels of permissive parenting. No statistically significant relationships were found between preschoolers’ gross motor skills and their digital game addiction tendency, nor was any significant relationship found between parents’ authoritative, authoritarian, or permissive parenting styles and their children’s gross motor skills. Likewise, there was no significant relationship between the children’s addiction tendency and their parents’ democratic parenting style. However, positive and significant relationships were identified between the overprotective parenting style and children’s gross motor skills, and between the authoritarian and permissive parenting styles and children’s digital game addiction tendency. Finally, a positive and statistically significant correlation was found between the overprotective parenting style and one addiction sub-dimension, constant play.

Although the participating children’s digital game addiction tendencies were low, every case of such addiction is potentially tragic, and parents, teachers, and policy-makers should all bear in mind that parenting style is a crucial factor in influencing children’s digital actions [95]. In the current study, while no significant relationship was found between children’s digital game addiction tendencies and the adoption of the democratic parenting style in their households, such tendencies were positively and significantly correlated with the authoritarian and permissive parenting styles. Parents and carers should carefully guide children’s screen usage and promote increased interaction with their surroundings and other people [63], and intervention programs could usefully be developed to increase parents’ awareness of this issue.

Regarding the observed low gross motor development of the children sampled in this study, it is worth mentioning that both structured and unstructured physical exercise therapies have been found to enhance general motor development, particularly in locomotion and object control [98]. The impact of these interventions—which include balance, coordination, strength, endurance, body awareness, posture, hand–eye coordination, finger and hand muscle strengthening, and two-hand coordination—on the gross motor development of Turkish preschoolers should be evaluated in future research. Also, Jia et al. [99] reported a significant correlation between parental attitudes towards physical education and improvement in their children’s motor skills. Therefore, researchers should develop and test intervention programs for parents emphasizing the positive effects of physical activity on their children’s physical health (obesity prevention, bone development, cardiovascular health), mental health (stress reduction, increased self-confidence, development of social skills) and academic success.

A limitation of this study is that various demographic characteristics of the children including age, sex, and birth order were not included in its analyses, yet could have had important effects on their gross motor competencies [8]. In further studies of children’s digital game addiction tendencies, their gross motor skills and their parents’ parenting styles should take account of such characteristics. Additionally, the current study did not include measurement sensitivity or possible mediating variables, which are limitations that should be addressed in future research.

## Figures and Tables

**Table 1:** Participant characteristics.

Variable		n	%	Mean
Height (cm)		252	100	110.94
Weight (kg)		252	100	21.24
Child’s Gender	Girl	126	50	
	Boy	126	50	
Child’s Age, Years	5	147	58.3	
	6	105	41.7	
Number of Siblings	0	30	30.6	
	1	77	43.3	
	2–3	109	13.1	
	4–5	33	1.2	
	6+	3	11.9	
Mother’s Highest Educational Attainment	Not Literate	21	8.3	
	Elementary School	50	19.8	
	Middle School	48	19.0	
	High School	63	25.0	
	Bachelor’s Degree	62	24.6	
	Postgraduate Degree	8	3.2	
Father’s Highest Educational Attainment	Not Literate	8	3.2	
	Elementary School	24	9.5	
	Middle School	38	15.1	
	High School	93	36.9	
	Bachelor’s Degree	75	29.8	
	Postgraduate Degree	14	5.6	
Mother Active in Sports	Yes	32	12.7	
	No	220	87.3	
Father Active in Sports	Yes	36	14.3	
	No	216	85.7	
Child Has Own Room	Yes	165	65.5	
	No	87	34.5	
Child Has Own Internet Access	Yes	55	21.8	
	No	197	78.2	
Child’s Daily Technology Usage Time, Hours	0	27	10.7	
	Up to 3	180	71.4	
	>3, <5	35	13.9	
	5+	10	4.0	
Mother’s Employment Status	Not Working	209	82.9	
	Working	43	17.1	
Father’s Employment Status	Not Working	22	8.7	
	Working	230	91.3	
Parents’ Marital Status	Married	241	95.6	
	Divorced	7	2.8	
	Widowed	4	1.6	
Child’s Duration of Preschool Education, Years	<1	145	57.5	
	1	65	25.8	
	2	42	16.7	

**Table 2:** Descriptive statistics.

Test for Gross Motor Development, 2nd edn.	Min.	Max.	Mean	Std. Deviation
Locomotor Skills	1.00	32.00	8.77	4.42
Object Control Skills	1.00	20.00	7.69	4.19
Total TGMD-2	1.00	38.00	16.30	7.21
**Digital Game Addiction Tendency**				
Dissociation from Life	7.00	35.00	15.97	7.29
Conflict	5.00	25.00	12.60	5.61
Constant Play	5.00	24.00	11.55	5.04
Reflection on Life	3.00	15.00	7.65	3.17
Total	20.00	97.00	47.77	18.68
**Parenting Style**				
Democratic	33.00	85.00	73.33	9.42
Authoritarian	11.00	55.00	22.75	8.90
Overprotective	20.00	45.00	35.33	5.87
Permissive	9.00	45.00	21.92	7.33

**Table 3:** Spearman’s rho results, relationship between preschoolers’ gross motor skills and their digital game addiction tendency.

		Locomotor Skills	Object Control Skills	Total TGMD-2
Dissociation from Life	r	−0.002	0.016	−0.013
	*p*	0.979	0.796	0.841
Conflict	r	0.001	0.008	−0.005
	*p*	0.993	0.897	0.936
Constant Play	r	−0.024	−0.002	−0.023
	*p*	0.702	0.980	0.721
Reflection on Life	r	−0.069	0.000	−0.035
	*p*	0.278	0.996	0.582
Total DGAT	r	−0.023	0.006	−0.021
	*p*	0.721	0.920	0.745

Note. r = correlation coefficient; *p* = significance level; TGMD-2 = Test for Gross Motor Development, 2nd edition; DGAT = Digital Game Addiction Tendency Scale.

**Table 4:** Spearman’s rho results, relationship between preschool children’s gross motor skills, and the parenting styles of their parents.

Parenting Style		Locomotor Skills	Object Control Skills	Total TGMD-2
Democratic	r	0.003	0.024	−0.022
	*p*	0.967	0.710	0.733
Authoritarian	r	0.113	−0.044	0.034
	*p*	0.072	0.484	0.592
Overprotective	r	0.129	0.079	0.140
	*p*	**0.041**	0.214	**0.026**
Permissive	r	0.030	−0.051	−0.010
	*p*	0.641	0.422	0.878

Note. r = correlation coefficient; *p* = significance level (bold if significant); TGMD-2 = Test for Gross Motor Development, 2nd edition.

**Table 5:** Spearman’s rho results, the relationship between preschool children’s digital game addiction tendency, and the parenting styles of their parents.

Parenting Style		Dissociation from Life	Conflict	Constant Play	Reflection on Life	Total DGAT
Democratic	r	−0.033	−0.058	−0.038	0.055	−0.022
	*p*	0.598	0.357	0.549	0.384	0.726
Authoritarian	r	0.278 **	0.223 **	0.251 **	0.217 **	0.280 **
	*p*	**0.000**	**0.000**	**0.000**	**0.001**	**0.000**
Overprotective	r	0.048	0.089	0.157 *	0.060	0.100
	*p*	0.451	0.161	**0.013**	0.342	0.114
Permissive	r	0.143 *	0.132 *	0.156 *	0.225 **	0.175 **
	*p*	**0.023**	**0.037**	**0.013**	**0.000**	**0.005**

Note. r = correlation coefficient; *p* = significance level (bold if significant); DGAT = Digital Game Addiction Tendency Scale; * *p* <0.05, ** *p* <0.01.

## Data Availability

Data will be made available upon reasonable request. The data are not publicly available due to [ethical issues that may arise due to the privacy of young children].
